# APP signaling in Alzheimer’s disease

**DOI:** 10.18632/aging.101641

**Published:** 2018-11-13

**Authors:** Elena Anahi Bignante, Alfredo Lorenzo

**Affiliations:** 1 Instituto de Investigación Médica “Mercedes y Martín Ferreyra”, INIMEC-CONICET- Universidad Nacional de Córdoba, Córdoba, Argentina

**Keywords:** Amyloid β (Aβ), Amyloid β precursor protein (APP), Go protein, Gβγ complex, Gallein

A large body of evidence supports the Amyloid β (Aβ) cascade hypothesis underlying neurodegeneration in Alzheimer’s disease (AD). Although the mechanism by which Aβ induces neuronal dysfunction and death is still matter of debate, in the last two decades several groups have generated compelling evidence supporting a role of Amyloid β Precursor Protein (APP) as a bona fide receptor for Aβ that can trigger neurodegeneration [1].

Our initial discovery that APP binds Aβ fibrils and mediates its neurotoxic effect on neuronal cultures [2] was subsequently extended by reports showing that harmful effects of diverse Aβ-assemblies are APP-dependent. Recently, Wang and collaborators reported that both, Aβ-derived diffusible ligands (ADDL) and Aβ oligomers extracted from human AD brain, impaired long-term potentiation in an APP-dependent manner [3]. Furthermore, another group showed that intracranial infusion of Aβ oligomers impaired associative fear and spatial learning in WT mice but had no amnesic effect in APP-KO mice [4].

How APP mediates toxicity of Aβ-assemblies? Working in hippocampal neurons in culture we provided initial evidence that APP is a receptor for Aβ fibrils that mediates toxicity by activating Go signaling [5] Thereafter, Fogel and collaborators extended this observation showing that, in cultured neurons naturally secreted Aβ binds to APP, activating a Go protein signaling cascade that modulates presynaptic glutamate release in physiological conditions [6]. Interestingly, these authors also observed that preventing Aβ degradation by neprilysine inhibition further enhanced APP-Go signaling and glutamate release. All these observations strongly suggest that accumulation of Aβ in AD brain might trigger pathological activation of APP-Go signaling, leading to neuronal dysfunction. We recently published data further supporting this hypothesis [6]. We found that APP-dependent toxicity of Aβ fibrils is mediated by Gβγ complex signaling, and we also identified p38MAPK as a downstream target of Gβγ complex. Furthermore, we found that Aβ fibrils enhanced the interaction of APP and Go protein in dystrophic neurites of mature hippocampal cultures, suggesting that Aβ deposition triggers sustained APP-Go signaling. Consistent with this interpretation, we observed that Gallein, a specific inhibitor of Gβγ signaling, protected mature hippocampal cultures against Aβ-induced dystrophy and degeneration. The protective effect of Gallein was robust and effective against toxicity induced by different Aβ aggregates, suggesting that sustained over-activation of APP and Go/Gβγ complex signaling is a common pathological pathway for diverse toxic Aβ species. In addition, we also found that the protective effect of Gallein *in vitro* extended to several pathologic markers characteristic of AD, including somatodendritic localization of abnormally phosphorylated tau, dystrophic degeneration of axons and dendrites, loss of synapses and neuronal cell death [7].

Mechanistically, Gallein prevented Aβ-induced phosphorylation of p38-MAPK in mature neurons, indicating that this kinase is a downstream target of Gβγ complex. In fact, SB203580, a specific inhibitor of p38-MAPK, effectively prevented Aβ-induced redistribution of phosphorylated tau to the somatodendritic compartment. However, SB203580 exerted a partial protection against Aβ-induced dendritic dystrophy, suggesting that, besides p38-MAPK, other effectors downstream Gβγ might participate in dendritic dystrophy. Regardless of this, Gallein prevented the loss of synaptophysin/PSD95 puncta in Aβ-treated cultures, underscoring Gβγ inhibition as an effective intervention to preserve synapses. To test the role of APP-Go/Gβγ signaling *in vivo* we utilized the 3xTg-AD mice, which develop Aβ-related deficits in synaptic plasticity and memory performance. By using the novel object recognition task we found that intrahippocampal injections of Gallein were effective in reversing memory impairment. This behavioral observation together with our *in vitro* data indicate that sustained activation of APP/Go protein Gβγ-complex signaling triggered by toxic Aβ assemblies might play a critical role in neuronal dysfunction and degeneration in AD.

Compelling evidence indicates that Aβ peptides activate APP/Go signaling in both, physiologic and pathologic conditions. Activation of APP/Go signaling by Aβ monomers/dimmers is physiologically regulated by degradation and clearance of the peptide. However, pathologic species of Aβ (oligomers/fibrils) that are resistant to clearance induce persistent APP/Go signaling that causes neuronal dysfunction and degeneration. This perspective on the physio-pathological role of APP in AD brings novel putative targets for therapeutic interventions.
